# Disentangling the Impact of Point Defect Density and Carrier Localization-Enhanced Auger Recombination on Efficiency Droop in (In,Ga)N/GaN Quantum Wells

**DOI:** 10.1021/acsphotonics.3c00355

**Published:** 2023-07-19

**Authors:** R. M. Barrett, J. M. McMahon, R. Ahumada-Lazo, J. A. Alanis, P. Parkinson, S. Schulz, M. J. Kappers, R. A. Oliver, D. Binks

**Affiliations:** † Department of Physics & Astronomy & Photon Science Institute, University of Manchester, Manchester M13 9PL, U.K.; ‡ School of Physics, University College Cork, Cork T12 R5CP, Ireland; § Tyndall National Institute, University of Cork, Cork T12 R5CP, Ireland; ∥ Department of Materials & Metallurgy, University of Cambridge, Cambridge CB3 0FS, U.K.

**Keywords:** InGaN, efficiency droop, Auger recombination, localization, point defect density, light-emitting diode

## Abstract

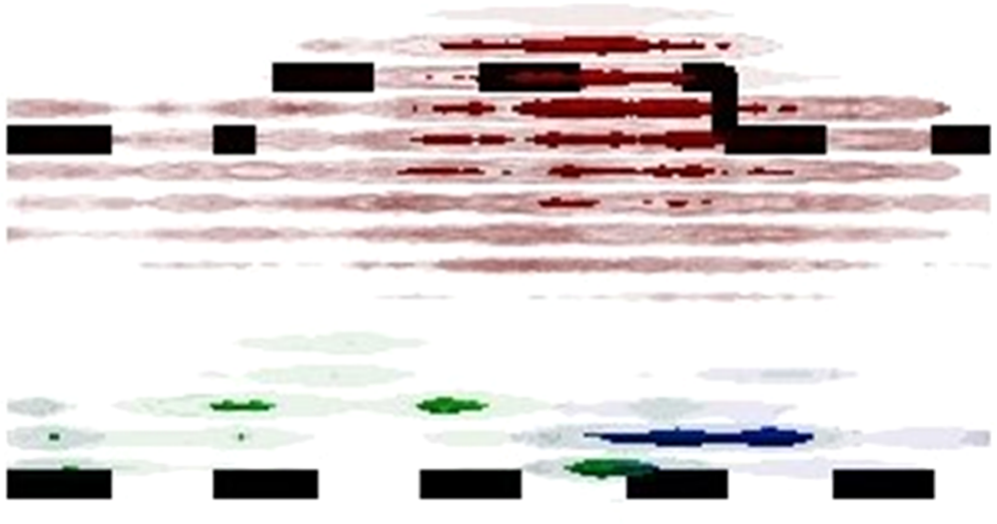

The internal quantum efficiency of (In,Ga)N/GaN quantum wells can surpass 90% for blue-emitting structures at moderate drive current densities but decreases significantly for longer emission wavelengths and at higher excitation rates. This latter effect is known as efficiency “droop” and limits the brightness of light-emitting diodes (LEDs) based on such quantum wells. Several mechanisms have been proposed to explain efficiency droop including Auger recombination, both intrinsic and defect-assisted, carrier escape, and the saturation of localized states. However, it remains unclear which of these mechanisms is most important because it has proven difficult to reconcile theoretical calculations of droop with measurements. Here, we first present experimental photoluminescence measurements extending over three orders of magnitude of excitation for three samples grown at different temperatures that indicate that droop behavior is not dependent on the point defect density in the quantum wells studied. Second, we use an atomistic tight-binding electronic structure model to calculate localization-enhanced radiative and Auger rates and show that both the corresponding carrier density-dependent internal quantum efficiency and the carrier density decay dynamics are in excellent agreement with our experimental measurements. Moreover, we show that point defect density, Auger recombination, and the effect of the polarization field on recombination rates only limit the peak internal quantum efficiency to about 70% in the resonantly excited green-emitting quantum wells studied. This suggests that factors external to the quantum wells, such as carrier injection efficiency and homogeneity, contribute appreciably to the significantly lower peak external quantum efficiency of green LEDs.

## Introduction

Light-emitting diodes (LEDs) based on (In,Ga)N/GaN quantum wells (QWs) are used for a variety of different lighting applications thanks to their excellent internal quantum efficiency (IQE), which can exceed 90% for blue-emitting devices at modest drive currents.^1^ However, the IQE can decrease significantly for longer emission wavelengths and with increasing carrier density. This latter phenomenon is known as “droop” and limits the use of (In,Ga)N/GaN-based LEDs in high-brightness applications such as vehicle headlights. Several mechanisms have been proposed to explain this droop behavior, including carrier escape,^2^ saturation of localization sites, which leads to an increased probability of carriers encountering point defects, and so undergoing nonradiative recombination,^3^ and intrinsic^4^ or defect-assisted Auger recombination.^5^ However, there remains significant debate over which of these processes, or which combinations of them, are most important for droop and thus over the best routes to reduce its effects, as summarized in ref (3).

The uncertainty over which are the most important mechanisms underlying droop is rooted in several challenges, both experimental and theoretical, that complicate the interpretation of data.^6−8^ On the experimental side, a challenging aspect is the accurate determination of the carrier density in the QW, *N*. This either relies on knowing how *N* depends on the excitation rate of the sample, which requires an understanding of the very recombination processes under study, or can be achieved using differential carrier lifetime measurements, which require simultaneous steady-state and pulsed excitation.^6^ Detailed discussion of different techniques to accurately determine carrier densities in III-N light emitters and the challenges connected to these experimental approaches can be found in ref (6).

Nevertheless, numerous attempts have been made to fit *N*-dependent *IQE* data and photoluminescence (PL) decay transients to basic descriptions of the key recombination processes.^8,9^ In particular, the “ABC” model has often been used, which is so-called because it assumes that the *N*-dependence of the IQE(*N*) in the QW is described by just three parameters (designated *A*, *B*, and *C*) corresponding to the coefficients for Shockley–Read–Hall (SRH), radiative, and Auger recombination, respectively, via
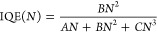
1In the simplest form of the ABC model, these three coefficients were assumed to be constants and evaluated by fitting them to experimental data. However, it has become clear from the experimental evidence gathered via measurements on a wide range of devices^6^ that these coefficients are not in fact constants but depend on *N*. Further, this conclusion was supported by the recognition that some important physics is not included in the simplest form of the ABC model, including the impact of the intrinsic polarization field in *c*-plane (In,Ga)N/GaN QWs (which affects the electron–hole wave function overlaps) on the recombination rate. These fields are screened with increasing *N*, resulting in an *N*-dependent *B* coefficient, for instance.^10^ Moreover, alloy fluctuations in (In,Ga)N/GaN QWs lead to strong carrier localization effects.^11^ These localization effects reduce the probability that carriers encounter defects and thus undergo SRH recombination; it has been suggested^12,13^ that they can become saturated at high *N* values so that carriers are exposed to more defects, and thus *A* increases with *N*. The value of the *C* coefficient can also be expected to be *N*-dependent since it too depends on the wave function overlap between carriers, which will change with increasing *N* as the polarization field is screened and localization sites are filled (with the latter effect also likely to cause *N-*dependence in the *B* coefficient, in addition to that produced by field screening).

Overall, the above discussion clearly highlights that (i) determining *N* accurately and (ii) knowing the *N*-dependence of *A*(*N*), *B*(*N*), and *C*(*N*) are crucial to gain insight into the importance of the various recombination process for droop in (In,Ga)N/GaN QWs, with theoretical insight especially important for (ii). While the impact of effects such as the intrinsic polarization fields on the radiative recombination rate can be studied with most commercially available software packages, the description of carrier localization effects cannot, including its effects on *A*(*N*), *B*(*N*), and *C*(*N*). In (In,Ga)N *bulk* systems, atomistic density functional theory (DFT)-based calculations have already demonstrated that the latter aspect can dramatically increase the Auger rate.^14^ However, the enormous growth in computational load with an increasing number of atoms in the simulation cell prohibits the use of these DFT calculations for (In,Ga)N QW systems.

The aim of this work is to disentangle the effect on the efficiency droop in (InG)aN/GaN QWs of point defect density and an Auger recombination rate enhanced by carrier localization. Full LED structures are complex, and their overall efficiency is affected by processes outside the active region and the uneven carrier distribution that can result from electrical injection.^6^ Since the focus of this study is the recombination processes within the active region, we investigate a simplified QW structure to enable a clearer understanding of them to be developed. Moreover, resonant PL measurements are used since they allow direct and broadly uniform excitation of the QWs with equal numbers of electrons and holes, enabling recombination processes in the active region to be studied separately from effects associated with carrier transport and capture and with inhomogeneous excitation. This combination of a simplified structure and resonant excitation has been previously shown to be an effective approach to understanding recombination in QWs.^15,16^ In this way, we investigate the effect of point defect density on droop by comparing measurements performed on a series of green-emitting *c*-plane (In,Ga)N/GaN QWs designed to have the same density of extended defects but different point defect densities, controlled by varying the growth temperature of the (In,Ga)N QW.^17^ The experimental studies are accompanied by theoretical studies that provide insight into the *N*-dependence of the carrier localization-enhanced radiative and Auger recombination rates. The atomistic tight-binding model accounts for (i) a microscopic description of the alloy disorder, (ii) the resulting fluctuations in strain and polarization fields, (iii) realistic length scales relevant to carrier localization effects, and (iv) the screening of the polarization field as carrier density increases in the (In,Ga)N/GaN QWs. We also present a novel method of comparing experiments and calculations in which we determine the IQE at a particular carrier density *N* without requiring any prior understanding of the recombination processes and with only a single excitation source.

Our combined theoretical and experimental investigations show that, indeed, Auger recombination is the main driver behind the droop effect within (In,Ga)N/GaN QWs, and it will therefore be important to the efficiency of LEDs at high drive current densities. By studying the IQE of QWs designed to have different point defect densities, we conclude that localization-enhanced Auger recombination effects are already sufficient to account for droop and, at least for the wells studied here, defect-assisted processes are of secondary importance in the high *N* > 10^19^ cm^–3^ carrier density regime. Even though Auger recombination plays a significant role in the droop effect, *under resonant excitation*, we estimate a peak IQE of our green-emitting QWs of >70%. In contrast, when a wavelength corresponding to above band gap excitation is used, the same samples exhibit a peak emission efficiency of 40–50%.^17^ These results suggest that (i) droop in (In,Ga)N/GaN QWs is an intrinsic process, does not depend significantly on defect density within them, and so is best mitigated by approaches that reduce *N*, such as current spreading and increasing the number of QWs, and (ii) factors external to the active region, such as the efficiency and homogeneity with which carriers are injected in the QWs, are of similar importance, particularly for peak efficiency and therefore are a necessary focus of future efforts to improve the *EQE* of green-emitting LEDs.

## Results

**Figure 1:**
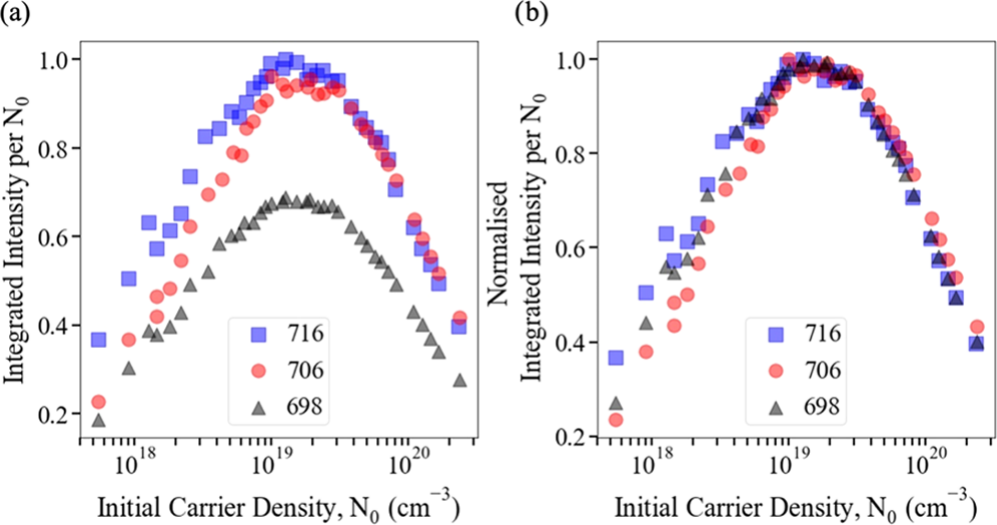
Integrated PL intensity divided by initial carrier density, *N*_0_, for the three samples: (a) relative amplitudes and (b) normalized to their peaks. Each sample is labeled by its growth temperature in °C.

Knowledge of the carrier densities excited during PL measurements is crucial if we are to compare our experimental results with calculations performed at fixed *N*. Under direct pumping of the QWs by ultrafast pulses, such as for the experiments described below, the initial value of carrier density, *N*_0_, can be calculated from the absorbed energy density of the pulses without prior knowledge of the carrier recombination dynamics because excitation occurs on a much faster timescale than recombination. Thus, relative emission efficiency can be characterized as the spectrally and time-integrated emission intensity per *N*_0_ and is plotted in Figure 1a for each of the green-emitting samples investigated in this study over three orders of magnitude in *N*_0_. This relative emission efficiency can be used as a proxy for IQE even though, as shown below, it is proportional to the IQE time-averaged over the decay of the carrier density. Each of the samples, described in detail in the Methods section, is labeled by its QW growth temperature, *T*_G_, in °C, which has previously been shown for these samples to inversely correlate with increased SRH recombination and so is consistent with a positive correlation with point defect density.^17^ Our previous studies also revealed that the emission efficiency measured in this way under identical experimental conditions can exhibit significant point-to-point variation across the sample,^18^ in a similar way to other reports that attributed it to the effect of step edges on local point defect density.^19^ Hence, while the data for a particular sample, which is taken at a fixed point on that sample, yields a reliable measure of the variation of IQE with *N*_0_ at that point, care must be taken with comparisons between samples. Nevertheless, the relative emission efficiency of the samples is consistent with expectations, i.e., at all of the *N*_0_’s studied, it increases with *T*_G_, consistent with the lower incorporation of point defects at higher growth temperatures. The relative performance of the three samples shown in Figure 1a is also consistent with the IQE values reported previously in the literature^17^ at lower excitation rates. However, what is most notable is the remarkable similarity of the behavior of the three samples at high *N*_0_ values (>1 × 10^19^ cm^–3^), which is most readily evident when comparing normalized versions of the droop curves, as shown in Figure 1b. In each case, the normalized efficiency increases initially as *N*_0_ increases before reaching a maximum at about *N*_0_ = 1 × 10^19^ cm^–3^; the efficiency then declines modestly until *N*_0_ = 3 × 10^19^ cm^–3^ before decreasing much more rapidly. There is a very close overlap of the *N*_0_ dependence of efficiency for each sample for *N*_0_ > 1 × 10^19^ cm^–3^, particularly the coincidence of the *N*_0_ values corresponding to maximum efficiency and to the onset of rapid efficiency decrease, despite their different *T*_G_’s. This suggests that the most important mechanisms responsible for droop do not depend on the point defect density of the QWs. This observation is explored further by comparing experimental data with the theoretical model.

**Figure 2:**
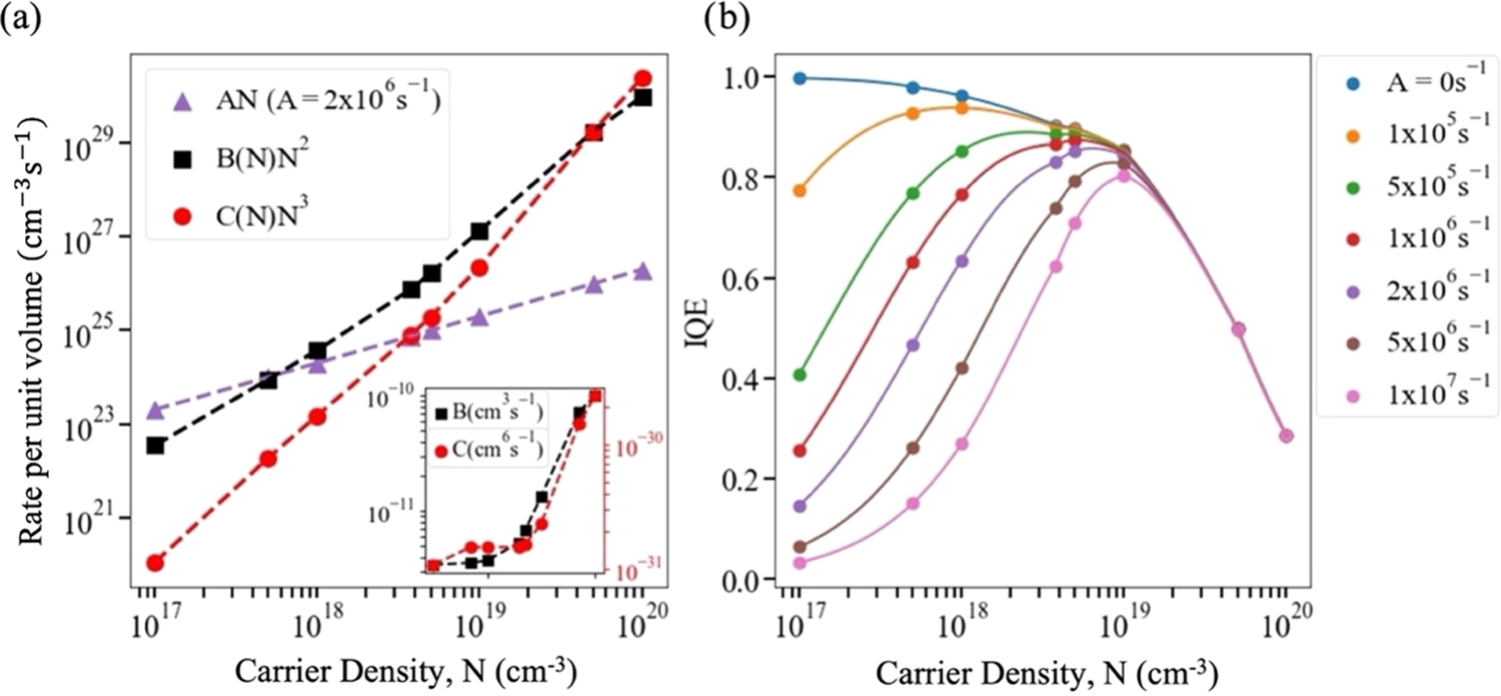
(a) SRH (AN), radiative (B(N)N^2^), and Auger (C(N)N^3^) recombination rates for a 15% indium, 3 nm wide (In,Ga)N/GaN QW, where B(N) and C(N) are the calculated values. The A coefficient is a sample value from the literature [18]. Inset: B(N) and C(N), averaged over two microscopic alloy configurations. (b) Calculated IQE curves for a range of SRH A coefficients.

While the point defect densities between the different samples vary, the samples—and indeed all (In,Ga)N QWs—have alloy fluctuation-induced carrier localization effects at the nanometer scale in common. The latter aspect is widely neglected when describing Auger recombination in (In,Ga)N-based QWs. Our model accounts for these effects at an atomistic level, and Figure 2a shows how the rate of each recombination process varies with *N*, where the calculated values of *B*(*N*) and *C*(*N*) and a typical *A* value^7,20,21^ have been used. Figure 2a demonstrates that for *N* > 1 × 10^19^ cm^–3^, SRH recombination is of secondary importance compared to the other processes. Figure 2b shows the IQE as a function of carrier density evaluated from eq 1 using the calculated *B*(*N*) and *C*(*N*) values shown in the inset of Figure 2a and a range of *A* values. Consistent with the experimental data displayed in Figure 1, the IQE becomes independent of the *A* values considered here for *N*_0_ > 1 × 10^19^ cm^–3^. The dependence of the IQE on *A* for smaller *N*_0_ values may explain why an apparent dependence of Auger recombination rate on defect density has been reported elsewhere^6^ since in that work *C*(*N*) was extracted by fitting to data extending to *N*_0_ ∼ 1 × 10^18^ cm^–3^.

To directly compare the experimental data with the calculated IQE, the appropriate *N* values must be used. In the model, *B*(*N*) and *C*(*N*) are evaluated for fixed values of *N*, and so the IQE(*N*) values calculated using eq 1 are also at fixed carrier density *N*. Experimentally, however, under pulsed excitation conditions, the simple quotient of the integrated PL intensity and *N*_0_, as in Figure 1, is proportional to the IQE(*N*) *averaged over N* as it decays from *N*_0_ to 0, as shown below. Instead, for comparison with theory, the IQE for a fixed value of *N* is required. This information can be determined as follows.

The evolution of *N*(*t*) following ultrafast pulsed excitation is described by
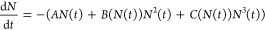
2and the emitted photon density, Φ, is proportional to the photon emission rate per volume, ϕ(*t*), integrated over time, where
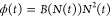
3Using eqs 2 and 3, Φ can be expressed as
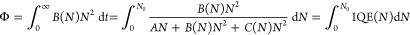
4Equation 4 thus confirms that dividing Φ(*N*_0_) by the initial carrier density, *N*_0_, corresponds to the IQE(*N*) averaged over *N* from 0 to *N*_0_. However, differentiating eq 4 with respect to *N*_0_ yields
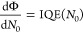
5i.e., the IQE at *N*_0_. *N*_0_ is controlled in this experiment by the absorbed photon fluence of the excitation pulse and corresponds to the carrier density that is set in the theoretical calculations.

**Figure 3:**
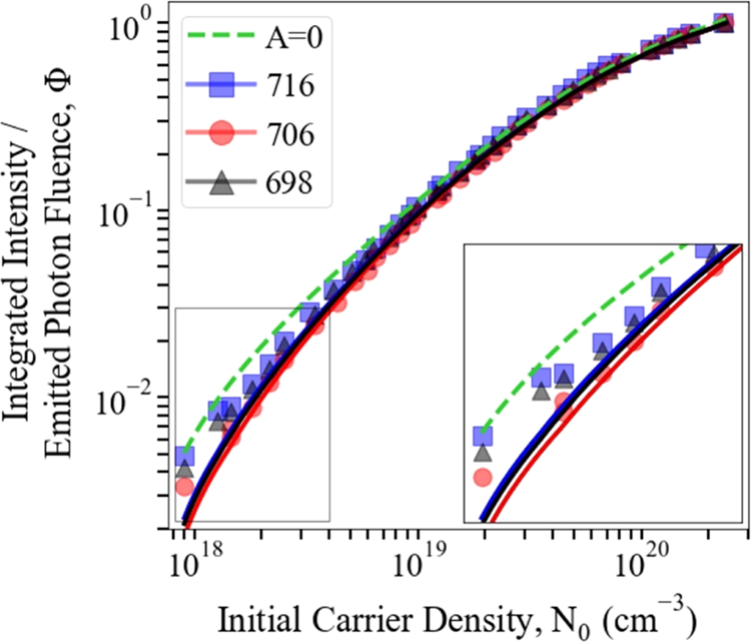
Integrated intensities (markers) and calculated emitted photon density, Φ (solid lines), evaluated using the calculated *B*(*N*_0_) and *C*(*N*_0_) values and *A* coefficients obtained from time-resolved measurements (see the main text). Each sample is labeled by its growth temperature in °C. The *A* = 0 s^–1^ case is included for comparison (dashed line). Inset: the expanded view of the low-carrier density region.

The normalized integrated intensities are plotted in Figure 3 for each of the samples; the plots are *not* significantly different between samples for *N*_0_ > 10^19^ cm^–3^ and diverge only slightly at lower *N*_0_ values. Also shown in Figure 3 is a normalized plot of Φ(*N*_0_) for the calculated *B*(*N*_0_) and *C*(*N*_0_), and *A* = 0 s^–1^. This is in excellent agreement with the experimental data for all samples for *N*_0_ > 10^19^ cm^–3^, and it too only differs slightly at lower *N*_0_. We emphasize that the calculated *B*(*N*_0_) and *C*(*N*_0_) values describe the experimental data excellently for *N*_0_ > 10^19^ cm^–3^ for the three different samples *without* any parameter fitting.

**Figure 4:**
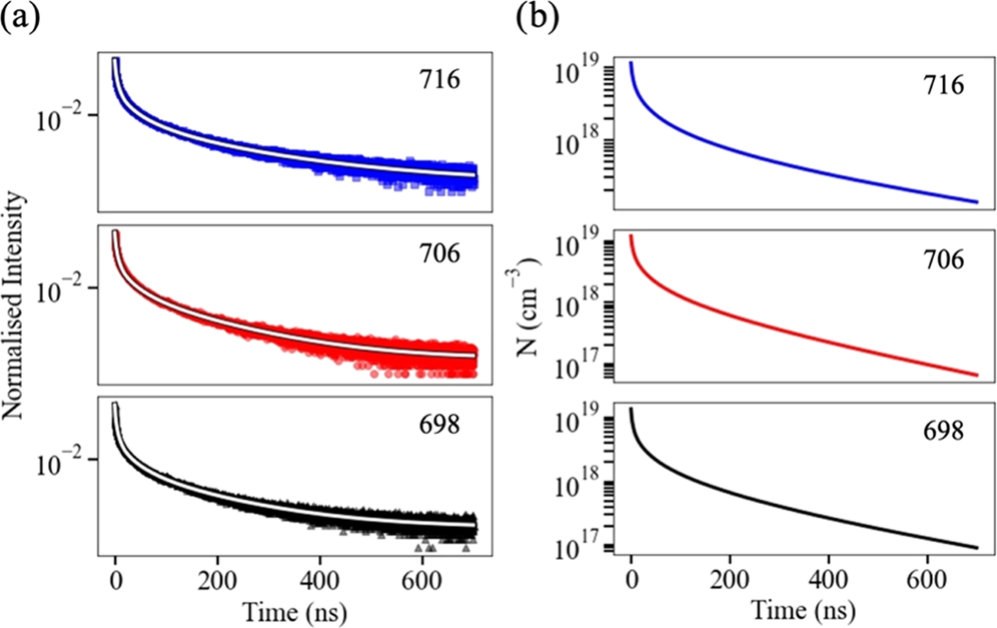
(a) PL decay transients (points) with fits to eq 3 (lines) for the three samples and (b) the corresponding calculated decay of the carrier density, *N*, found from eq 2. Each sample is labeled by its growth temperature in °C.

The weak dependence of this data on *A* demonstrates that SRH recombination is not an important recombination process at the *N*_0_ values corresponding to efficiency droop. However, it also means that using the calculated *B*(*N*) and *C*(*N*) in eq 4 and fitting to the data in Figure 3 does not constrain the *A* coefficients well for the samples studied here. The *A* values are somewhat better constrained by considering the PL decay transients shown in Figure 4a since, as Figure 4b shows, the tail of these extend to lower *N* values (*N*_0_ ∼ 10^17^ cm^–3^), corresponding to the regime in which SRH recombination is significant (see Figure 2). By inserting the calculated *B*(*N*) and *C*(*N*) into eqs 2 and 3, ϕ(*t*) may be fitted to the PL transients for each of the samples, as also shown in Figure 4a. In each case, the only fitting parameters are *A* and *N*_0_, the latter of which is allowed to vary since previous studies^22^ have shown that for high *N*_0_ the value of *N* can decrease significantly within the first 100 ps after excitation, i.e., on a timescale less than the time resolution of the experimental apparatus. This fit yields estimates for *A* in the range from 2 × 10^6^ to 4 × 10^6^ s^–1^, which is similar to previous reports.^7,20,21^ As noted above, a previous study^18^ of the spatial variation of the PL of these samples has shown that the intensity can vary by as much as 40% from point to point; however, nonetheless, this range of values may be regarded as broadly representative of this sample set. However, the spatial variability of *A* is not of significant consequence for this study since its emphasis is on *N* values corresponding to the droop regime, at which SRH recombination is negligible, as shown in Figures 1–3.

By inserting these *A* values into eq 4, Φ(*N*_0_) curves may be calculated for the three samples and are also shown alongside the experimental integrated intensities in Figure 3. In each case, the curves are, again, *not* significantly different from each other or the experimental data for *N*_0_ > 10^19^ cm^–3^, but all improve the agreement with their corresponding experimental data (symbols in Figure 3) at lower *N*_0_ compared to the curve calculated with *A* = 0 s^–1^.

**Figure 5:**
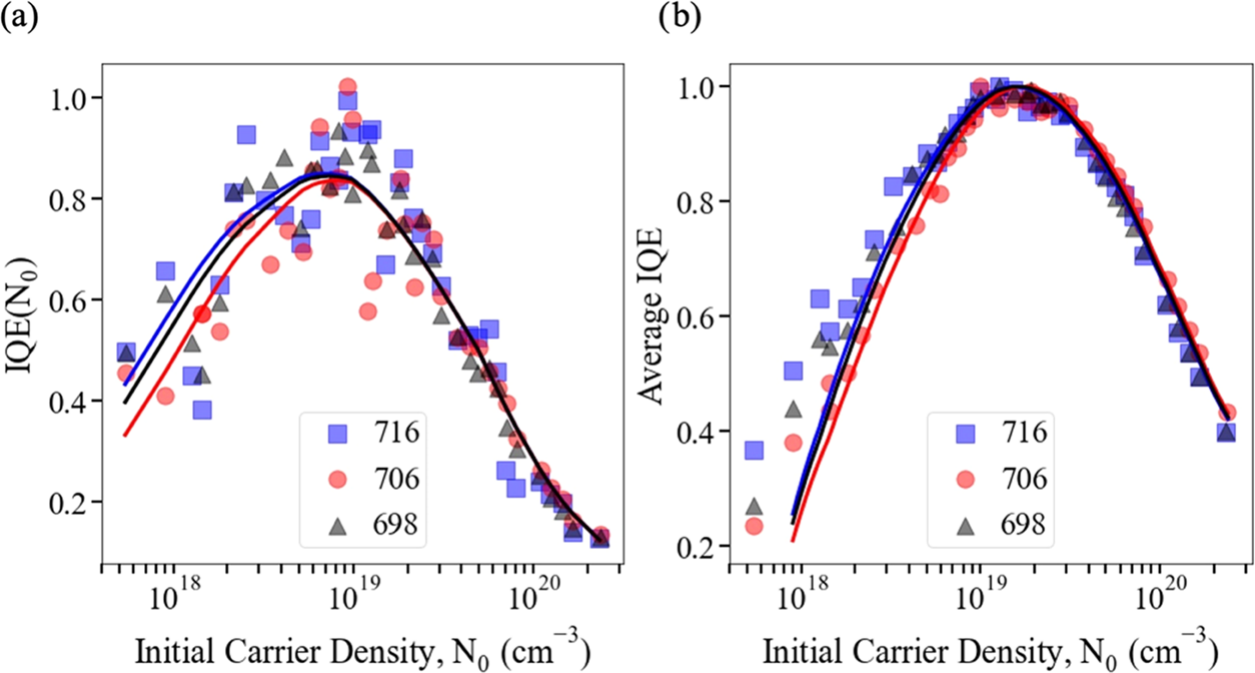
Comparison of calculated (lines) and experimentally measured (symbols) values of (a) IQE(*N*_0_) and (b) average IQE during a pulse. For (a), the derivative of the normalized integrated intensity shown in Figure 3 was smoothed using a Savitzky–Golay filter.^23^ For both (a) and (b), the experimental data has been scaled vertically to fit the calculated values since the measurements only determine relative, rather than absolute, IQE. Each sample is labeled by its growth temperature in °C.

*IQE*(*N*_0_) was evaluated for each of the three samples by eq 1 using the calculated *B*(*N*_0_) and *C*(*N*_0_) values and the *A* coefficients determined above. Figure 5a compares this calculated IQE(*N*_0_) to that determined from experimental data via eq 5, i.e., by finding the derivative of integrated intensity with respect to *N*_0_. Despite the additional scatter introduced to the data by the process of numerical differentiation, there is excellent agreement between the calculated and scaled experimentally determined IQE(*N*_0_). The average IQE over the decay of the pulse, i.e., Φ/*N*_0_, was also calculated using eq 4 and in Figure 5b was compared to the experimental average IQE, which is proportional to integrated intensity per *N*_0_. Again, there is a very good agreement between calculations and measurements. Thus, the experimental data is well described by a constant *A* value for each sample. Given the negligible contribution of SRH recombination at high *N*, an *N*-dependence of *A* cannot be ruled out, but it is clear any such *N*-dependence does not have a significant effect on the IQE or recombination dynamics in this range.

The calculations of IQE(*N*_0_) yield absolute values for the peak IQE, which are approximately 80% for each sample. We note that typical supercell configurations were employed to calculate the *B*(*N*) and *C*(*N*) values used above, but approximately 15–20% of the configurations studied may exhibit noticeably increased *C*(*N*) values.^24^ The peak IQE dropped to approximately 70% if average *C*(*N*) values, weighted by the fraction of configurations in which each *C*(*N*) value occurred, were used. If such high *C*(*N*) regions exist in the samples, then they *could* act as a “sink” region for carriers that are sufficiently mobile. However, since there is also good agreement between calculations and the average IQE data, which includes the contribution from carriers that have the time to redistribute within the QW, there is no evidence that this is a significant effect. Moreover, room-temperature IQEs similar to the peak IQEs described here have been reported previously for high-quality green-emitting QWs: when determined by resonantly excited differential carrier lifetime measurements, IQEs ranging from 85 to 70% were observed as the indium content was increased from 10 to 28%.^6,25^ As reported previously, the peak IQE for our samples obtained via the above band gap excitation was 40–50%,^17^ which is similar to the *EQE* values of 40–50% typically reported for green-emitting LED structures.^26^ In both cases, additional factors such as carrier injection efficiency, an uneven carrier distribution between QWs, and current leakage^27^ may further reduce device efficiency.

## Discussion

A rational approach to the design of QW structures to minimize droop was inhibited when it was unclear which of the possible mechanisms for droop was most important because good agreement between theory and experiment was difficult to achieve. For instance, if defect-assisted Auger recombination *was* most important, then the focus would have been on the minimization of the point defect density in the QW. However, good quantitative agreement between measurement and calculated IQE has now been demonstrated, showing that Auger recombination, enhanced by the localization of carriers but without any defect assistance, is sufficient to explain droop in the green-emitting QWs studied here. The *A* coefficient does determine the peak IQE, as shown in Figure 2, but for carrier densities ≳10^19^ cm^–3^, which correspond to significant droop, it has negligible impact on the IQE. Moreover, the peak IQE values of ∼70% reported here under resonant excitation for samples that are designed to vary in point defect density and elsewhere^6,25^ show that there is limited scope for further improvement of *maximum* recombination efficiency within the active region of green-emitting QWs.

However, to achieve efficient high-brightness green LEDs, a high-peak IQE is insufficient if this corresponds to a carrier density, *N*_peak_, significantly less than the operating value. It is thus important to be able to reduce the carrier density *N* produced for a certain drive current density, *j*, toward *N*_peak_. This may be achieved by utilizing QWs grown along alternate crystal directions or in alternate phases that have lower recombination lifetimes, τ, since *N* = *j*τ/*et*_QW_, where *t*_QW_ is the QW thickness. For instance, QWs produced from *m*-plane wurtzite^28^ or zincblende GaN^29^ have sub-nanosecond recombination lifetimes rather than lifetimes of 10s or 100s of nanoseconds as typically found for *c*-plane wurtzite green-emitting QWs.^17^ However, no significant reduction in droop has yet been observed in nonpolar QWs^30,31^ and droop measurements in zincblende GaN QWs have yet to be reported. The increase in wave function overlap produced by colocalization of electrons and holes in such systems may increase the Auger recombination rate sufficiently to negate the benefit of reduced carrier density.^32^

An active region with a high maximum recombination efficiency at the operating carrier density is a necessary, but not sufficient, requirement for achieving high-brightness green LEDs. Given the above observations and the much lower (40–50%) values of IQE reported for *nonresonant* excitation of the QWs^17^ and for external quantum efficiency (EQE) measured for green-emitting LED structures,^26^ this suggests that there is more scope for increasing *peak* device efficiency by focusing on aspects other than recombination within the active region. For instance, improving the efficiency and homogeneity of carrier injection into the active region or reducing current leakage is likely to yield greater increases in EQE. This work also indicates that the most productive route to reducing droop is to target active region designs that reduce carrier density in individual QWs, such as the increased QW area or QW number or a more even distribution of carriers between QWs, rather than trying to reduce SRH recombination in the active region.

## Conclusions

Our combined theoretical and experimental investigations on green-emitting (In,Ga)N/GaN *c*-plane QW systems grown at different temperatures show that carrier localization-enhanced Auger recombination is sufficient to explain droop without defect-assisted contributions. At high carrier densities, *N*, both the IQE at a particular *N* and the time-averaged IQE following pulsed excitation are not significantly affected by the growth temperature, which suggests that defect-assisted processes are of secondary importance at high carrier densities. We find excellent agreement between theory and experiment, and the calculated *B*(*N*) and *C*(*N*) are sufficient to describe the decay dynamics, with the *A* coefficient only becoming significant at lower *N*’s.

Since defect-assisted effects are negligible above a carrier density of ∼10^19^ cm^–3^, reducing the point defect density in QW devices operating at *N* ≳ 10^19^ cm^–3^ will not significantly reduce efficiency droop. Instead, the significant decrease from the peak IQE value that occurs due to Auger recombination as *N* increases beyond ∼10^19^ cm^–3^ is best mitigated by designing active area architectures that reduce *N* in individual QWs, such as increasing the total number of QWs or the QW area and evenly distributing carriers between the QWs. The *peak* IQEs calculated here for our samples (70%) and the steady-state IQEs measured by PL techniques in refs (6, 25) are much higher than *peak* EQEs reported for LED devices,^23^ which suggests that effort should also be focused on improving carrier injection efficiency and the homogeneity of the distributions of carriers between QWs.

## Methods

### Experiment

A series of three green-emitting (In,Ga)N/GaN MQW samples were grown via metal–organic chemical vapor deposition (MOCVD) in a 6 × 2 in. Thomas Swan close-coupled showerhead reactor at a pressure of 300 Torr using trimethylindium (TMI), trimethylgallium (TMG), and ammonia (NH_3_) as precursors and nitrogen as the carrier gas. The five-period-MQW structures were grown on GaN pseudosubstrates, consisting of ∼4 μm of GaN on (0001) sapphire substrates. The samples were not intentionally doped. For each of the three samples, a different QW growth temperature was chosen, while the GaN barriers were grown at a higher, more optimal temperature using the two-temperature (2T) growth method.^33^ The recombination efficiency of similar LED structures in which the active region was grown by this method has been determined to be 65% previously by electroluminescent measurements.^33^ X-ray diffraction (XRD) was used to characterize the thickness and composition of the MQW structure by performing an ω–2θ scan along the symmetric (002) reflection. However, due to the presence of gross well width fluctuations (GWWFs), XRD only tells us the combined width of the QW plus barrier periods and the average indium composition of the periods. The nominal thicknesses of the (In,Ga)N QWs and GaN barriers (including the topmost one) were 3 and 7 nm, respectively, and the indium content in the QWs was thus estimated to be 17 ± 2%, matching the value assumed in the model to within the limits of the measurements. The chosen *T*_G_ and XRD results for each sample, termed 716, 706, and 698 throughout the rest of this paper, are summarized in Table 1. The growth temperature, *T*_G_, of the QW has been shown to affect the nonradiative recombination rate at low excitation densities,^17^ such that a lower *T*_G_ results in a shorter 300K PL decay lifetime and lower IQE. In ref (17), Hammersley et al. attributed this to an increase in the density of point defects at lower *T*_G_’s. It should be noted that a recent detailed transmission electron microscopy study^34^ on these samples has suggested that although the spatially averaged structural parameters of the samples are very similar, there may be slight differences in the nanoscale structure of the QWs, with QWs grown at higher temperatures exhibiting a greater density of GWWFs. However, these GWWFs are expected to reduce the vulnerability of the QWs to recombination at point defects so that the Hammersley et al. interpretation of the PL data still holds.

**Table 1:** Summary of the QW Growth Temperatures, Period, and Indium Content for the Samples Studied

sample	QW growth temperature, *T*_G_ (°C)	period width (nm)	period In content (%)
716	716	9.9 ± 0.2	5.1 ± 0.5
706	706	9.9 ± 0.2	5.2 ± 0.5
698	698	10.0 ± 0.2	5.1 ± 0.5

For the optical measurements, the QWs were directly excited at room temperature by 150 fs pulses from a Ti:sapphire laser source, operating at a repetition rate of 250 kHz and frequency-doubled to generate an excitation wavelength of 400 nm. The laser beam was defocussed to an excitation spot size of 20 μm, and a neutral density (ND) filter wheel was used to vary the excitation density between 1.5 and 660 μJ cm^–2^ pulse^–1^. The laser spot had a Gaussian profile, so luminescence was only collected from a 5 μm spot at the center of the excitation area to ensure that only emission from a region of near-uniform excitation was detected. The light emitted by the sample was collected by an optic fiber and directed into an Ocean Optics spectrometer. Time-resolved measurements were obtained using time-correlated single-photon counting (TCSPC) at an excitation density of 330 μJ cm^–2^ pulse^–1^. Emission was collected over the first 700 ns of the decay and spectrally integrated across the peak. The experimental initial excitation density can either be expressed as an areal carrier density, which is equivalent to the absorbed photon fluence, or for direct comparison with the calculated carrier densities, *N*_0_, as a volume density by dividing the areal density by the QW thickness.

### Theory

A full description of the atomistic theoretical framework used in the evaluation of the radiative and Auger recombination coefficients can be found in refs (11, 24, 34, 35); here, only the main aspects are summarized. To obtain the electronic structure required for the radiative and Auger rate calculations discussed above, we use a nearest-neighbor, sp^3^ tight-binding (TB) model, which takes input from a valence force field and local polarization theory approach. In doing so, the framework accounts for random alloy fluctuations and connected carrier localization effects on an atomistic level. As described in our previous work,^36^ the model has been parameterized and benchmarked against hybrid-functional (HSE) density functional theory (DFT) and experimental literature data. Moreover, our atomistic theoretical framework has shown good agreement with experimental data on (In,Ga)N/GaN QWs, highlighting that it captures alloy-induced carrier localization effects accurately.^37^

All calculations have been performed on a supercell with approximately 82,000 atoms. For the (In,Ga)N QWs, a random alloy distribution has been assumed. A well width of approximately 3 nm is used in the calculations, and the indium content of the well is 15%. Both settings are in good agreement with the experimental data above. Changes in the electronic structure and thus the recombination rates due to screening of the electrostatic built-in potential with increasing carrier densities in the QW are accounted as follows: First, at an atomistic level, using our developed local polarization theory,^36^ the electrostatic built-in potential arising from spontaneous and piezoelectric polarization has been evaluated. As already mentioned above, this fully three-dimensional calculation includes alloy disorder-induced fluctuations in the potential. As an atomistic, three-dimensional, fully self-consistent calculation to account for screening the built-in field with increasing carrier density is numerically prohibitive, we employ a method similar to refs (38) and perform a self-consistent one-dimensional calculation using directly input from the atomistic calculation (e.g., average strain). This analysis yields the degree to which the built-in field is screened at a given carrier density. The information is subsequently used in the atomistic calculation to adjust the intrinsic built-in field accordingly. We find that the onset of the importance of screening the built-in field aligns very well with recently experimental studies on similar systems.^6,39^ Overall, we note that defect-related effects are not taken into account in the model.

## Data Availability

The data underlying this study are openly available in the University of Manchester Repository at DOI 10.48420/22801913.
